# Special Issue “Targets, Tracers and Translation Novel Radiopharmaceuticals Boost Nuclear Medicine”

**DOI:** 10.3390/ph12030111

**Published:** 2019-07-18

**Authors:** Gerald Reischl

**Affiliations:** Department of Preclinical Imaging and Radiopharmacy, Eberhard Karls University of Tübingen, Röntgenweg 15, D-72076 Tübingen, Germany; gerald.reischl@med.uni-tuebingen.de

This is the fourth Special Issue in *Pharmaceuticals* within the last six years dealing with aspects of radiopharmaceutical sciences. It demonstrates the significant interest and increasing relevance to ameliorate nuclear medicine imaging with PET or SPECT, and also radiotherapeutical procedures.

Numerous targets and mechanisms have been identified and have been under investigation over the previous years, covering many fields of medical and clinical research. This development is well illustrated by the articles in the present issue, including 13 original research papers and one review, covering a broad range of actual research topics in the field of radiopharmaceutical sciences.

Imaging in oncology still is the most important area, demonstrated by five papers, dealing with the identification of a target of interest, development of a radiopharmaceutical (tracer) to image the target, followed by first evaluations in vitro (cell experiments) or in vivo in small animals. Hypoxia is a characteristic property that can be found in various tumor tissues and therefore has been an interesting target for imaging for many years. Maier et al. linked deoxyribose to 2-nitroimidazole a common hypoxia pharmacophore, thereby mimicking a nucleoside, labeled the molecule with ^18^F (PET) and investigated the uptake pattern in a tumor mouse model. They found a faster uptake of the tracer compared to the well-known [^18^F]FMISO, which was attributed to the involvement of active transport mechanisms by nucleoside transporters. More recently, various receptors specifically expressed by tumor cells, became targets for molecular imaging. Here, Kaloudi et al. chose the gastrin-releasing peptide receptor (GRPR) as target and developed two ligands, labeled with ^99m^Tc for SPECT and evaluated their biological behavior in prostate cancer cells and in mice. Uptake in GRPR-expressing lesions could be drastically enhanced by neprilysin inhibition. The cholecystokinin-2 receptor (CCK2R) was the target in the study by Klingler et al. Their HYNIC-conjugated minigastrin analogs, also labeled with ^99m^Tc, showed a high biostability and a high tumor uptake in tumor bearing mice, which make the derivatives promising SPECT ligands. To generally accelerate and enhance tumor uptake of tracers two concepts have been in discussion for some time. Pretargeting may be advantageous for tracers that show slow biokinetics, like antibodies. The antibody is injected first, after the time necessary to bind to its target, in a second injection a radiolabeled ligand that shall selectively bind to the antibody (e.g., via click chemistry) is administered. With this rationale, long uptake times of radiolabeled antibodies (long-lived isotopes necessary) may be overcome, also reducing radioactive doses for patients. To improve binding of a ^68^Ga-labeled ligand to the pretargeted antibody, Summer et al. synthesized ligands with one to three 1,2,4,5-tetrazines for binding to the antibody. They could show in vitro and in vivo that the binding capability was improved with an increasing number of tetrazines at the ligand. Another concept with the intention to improve tumor uptake of tracers is the design of bivalent/bispecific ligands, in the case when two different receptors are present in a certain tumor type. Vall-Sagarra et al. could demonstrate the value of this concept by developing ^68^Ga-labeled peptidic ligands to address two different receptors in breast cancer (neuropeptide Y receptor and GRPR). Tumor uptake was considerably higher compared to the corresponding monovalent tracers.

In neuro imaging, for more than two decades numerous tracers have been developed to image various brain receptors. Challenges in these efforts have been the in vivo stability of compounds, their ability to cross the blood-brain-barrier and especially their specificity versus the different types and subtypes of receptors. Receptor binding of tracers may also be influenced by, e.g., the presence of the endogenous receptor ligands. Müller Herde et al. investigated the receptor binding of [^18^F]PSS232, a derivative of the longer known [^11^C]ABP688, a ligand for the metabotropic glutamate receptor 5 (mGluR5), in dependence of glutamate levels. Both in vitro autoradiography on rat brain slices and in vivo PET showed no change of tracer binding upon drug-induced altering of glutamate levels. They concluded that in contrast to [^11^C]ABP688, [^18^F]PSS232 cannot be used to measure fluctuations of glutamate levels, but is useful to image mGluR5 occupancy. In another study by Tanzey et al. a novel PET tracer was synthesized and in vivo evaluated for targeting the colony stimulating factor 1 receptor (CSF1R), which is involved in both neuroinflammation (microglia) and inflammation in the periphery (macrophages). Although it turned out that in rodent and non-human primate PET brain uptake was very low, the tracer may still be useful for imaging of peripheral inflammation. Further, imaging in immunology, whether it is inflammation or infection, has gained increasing interest over the last years.

The above described studies within this special issue consistently demonstrate that besides the radiochemical aspects the in vitro and in vivo evaluations are an integrative part already at an early stage in the development of a new tracer. This trend within the last decade was strongly supported by a rapidly increasing availability of a large variety of animal and in vitro models and on the other hand by the broad dissemination of small animal imaging using dedicated PET but also SPECT scanners. Besides ^18^F and ^11^C, ^68^Ga has made its way to the clinics and is regularly used in different tracers. Other longer-lived PET isotopes like ^64^Cu or ^89^Zr are still more research focused. However, as shown by the contributions in this issue research regarding development of SPECT tracers, labeled with ^99m^Tc or iodine isotopes (^131^I, ^123^I) is also substantial, not least due to the possibility of quantification with modern SPECT/CT systems or regarding the application of ^99m^Tc tracers in radio-guided surgery (see Klingler et al.).

Development and optimization of labeling strategies for SPECT tracers was the aim of three other studies, presented in this issue. Durante et al. could show systematically that for ^131^I-labeling Chloramine T has advantages over Iodo-Gen^®^, due to shorter reaction times and solubility in aqueous medium. For the first time, two tirapazamine derivatives were ^131^I-radioiodinated by Elsaidi et al. Tirapazamine is known as radiosensitizer of hypoxic tissue. Reaction parameters were optimized and could be controlled in a way to obtain either the one or the other product, which are now available to be evaluated for hypoxia imaging. For imaging of mannose receptors expressed by lymph node macrophages, Boschi et al. successfully developed a strategy for ^99m^Tc-labeling of mannose-dextran derivatives, using the highly stable ^99m^TcN synthon.

Radiopharmaceutical sciences and nuclear medicine are not limited to high-specific molecular imaging but are also fields of expertise in therapy and as a consequence ideal for theranostic approaches. The prostate-specific-membrane-antigen (PSMA) in prostate cancer has been a major focus in nuclear medicine over the last years, numerous tracers have been developed for imaging and therapy. For this indication, the most commonly used therapeutic isotope today is ^177^Lu, but also alpha-emitters gain increasing interest. For a better mechanistic understanding Tönnesmann et al. systematically investigated the uptake characteristics in salivary glands of the most prominent endoradiotherapy tracer [^177^Lu]Lu-PSMA-617 in vitro. Accumulation in salivary glands could be attributed to both specific and non-specific uptake mechanisms. These results are of high value for a future design of radiopharmaceuticals targeting PSMA. Basaco et al. developed ^177^Lu-labeled girentuximab, an antibody targeting carbonic anhydrase IX, expressed on most renal cancer cells. The authors optimized labeling conditions and found increasing in vitro antigen affinity and immunoreactivity with a decreasing number of ^177^Lu binding chelators DOTA per antibody.

Typically, radiopharmaceutical sciences are embedded in a clinical environment. All basic research in radiochemistry or preclinical radiopharmaceutical development finds its justification and needs the perspective of a consequent translation of the results into clinical application in nuclear medicine. The statement “a mouse is not a man” is trivial and translation “from bench to bedside” must be the driving force. Regulatory requirements and especially GMP have become a challenge to be faced and handled, but nevertheless cannot be showstoppers. A tracer that already made its way into clinical application for cardiac imaging is [^11^C]meta-hydroxyephedrine ([^11^C]mHED). On the other hand, this is a good example that translation “from bench to bedside” is not a uni-directional process. As production is challenging and specific activity and impurities from the precursor obviously affect image quality, Vraka et al. describe in their study their way back “from bedside to bench” to investigate the various influences. After optimization of the synthesis, the improved product was again evaluated with PET imaging in rats and later in humans, proving little effect of specific activity on image quality. In a review, Schirrmacher et al. summarized the current status in development of PET tracers to image tropomyosin receptor kinases (Trk), which are involved in neurological disorders, but also in tumorigenesis in a variety of human cancers. Trk inhibitors have been developed as PET probes and some were already translated to human application.

The present special issue on radiopharmaceutical developments of course can only reflect a limited overview of the actual broad spectrum of trends in this field. In general, possible contributions of radiopharmaceutical sciences and nuclear medicine imaging to a personalized medicine have been shown in the past and even seem to be broader in the future. Imaging sciences today are regarded as an integral part of clinical research. With new therapies becoming more complex and specific, imaging together with, e.g., metabolomics procedures, histology and advanced data analysis is a most valuable tool in such multi-modal approaches, providing molecular and functional profiles for therapy planning and monitoring.

